# Smartphone-Based Electrochemical Potentiostat Detection System Using PEDOT: PSS/Chitosan/Graphene Modified Screen-Printed Electrodes for Dopamine Detection

**DOI:** 10.3390/s20102781

**Published:** 2020-05-14

**Authors:** Xiaoyan Shen, Feng Ju, Guicai Li, Lei Ma

**Affiliations:** 1 School of Information Science and Technology, Nantong University, Nantong 226019, China; xiaoyansho@ntu.edu.cn (X.S.); jufeng199412@163.com (F.J.); 2 Co-Innovation Center of Neuroregeneration, Nantong University, Nantong 226019, China; 3 Key Laboratory of Neuroregeneration of Jiangsu and Ministry of Education, Nantong University, Nantong 226001, China; gcli1981@ntu.edu.cn

**Keywords:** dopamine, conducting polymer, graphene, smartphone, electrochemical detection

## Abstract

In this work, a smartphone-based electrochemical detection system was designed and developed for rapid and real-time detection of dopamine (DA). The system included a screen-printed electrode (SPE) used as a sensor, a hand-held electrochemical potentiostat and a smart phone with a specially designed app. During the detection period, the SPEs modified with poly(3,4-ethylenedioxythiophene) (PEDOT), chitosan (CS) and graphene (G) were used to convert and amplify the electrochemical reaction signals. The electrochemical potentiostat was used to generate excitation electrical signals and collect the electrical signals converted from the sensor. The smartphone—connected to the detector via Bluetooth-was used to control the detector for tests, further process the uploaded data, and plot graphs in real time. Experimental results showed that the self-designed sensing system could be employed for highly selective detection of DA in the presence of interfering substances such as ascorbic acid (AA) and uric acid (UA). CV was carried out to characterize the electrochemical properties of the modified SPEs and the electrochemical behaviors of DA on the modified SPEs. Finally, according to the analysis of DPV responses of DA, the system could detect DA with a detection sensitivity of 0.52 ± 0.01 μA/μM and a limit of detection of 0.29 μM in the linear range of DA concentrations from 0.05 to 70 μM.

## 1. Introduction

Dopamine is a key neurotransmitter secreted by the hypothalamus and pituitary gland [1]. While it directly affects people’s emotions, its secretion is also affected by mental factors. Numerous studies have demonstrated that neurological diseases, such as schizophrenia and Parkinson’s, can occur and develop at a low level of dopamine [2]. The traditional standard regimen for Parkinson’s disease is to supplement dopamine or levodopa to maintain a stable concentration of dopamine in patient’s brain [3], while it is always accompanied with a large number of side effects such as dyskinesias and emotional uncontrollability, which may be due to the fact that the traditional therapies supplemented patients with excessive dopamine or levodopa, resulting in the activation of non-motor neurons [4,5]. Hence, it is vital to design and develop a rapid, portable and real time biologic dopamine concentration detection system because that it could not only provide doctors with timely data to improve the efficiency of diagnosis and treatment, but also reduce side effects resulted from over-supplement of dopamine.

Various methods, such as high-performance liquid chromatography [6], surface plasmon resonance [7], colorimetry [8], chemiluminescence [9], spectroscopy [10] and electrochemistry [11], have been developed and utilized for dopamine detection. Among them, electrochemical methods, such as cyclic voltammetry [12] (CV), differential pulse voltammetry [13] (DPV) and electrochemical impedance spectroscopy [14] (EIS), etc., have been widely attention to and used due to their simple operation, fast testing speed and other advantages. As an electroactive compound, dopamine will coexist with chemicals in blood that possesses similar electrochemical properties with it, such as ascorbic acid and uric acid. It will cause confusion on their electrochemical identification owing to the similarity in their properties [15]. In order to improve the selectivity, sensitivity and detection range of dopamine, carbon nanomaterials [16] and conductive polymers [17] have been used to modify electrodes. As a new type of carbon-based nanomaterial, G has advantages in basic properties such as large specific surface area, outstanding electron transport performance, high mechanical strength, good chemical stability and exhibits good biocompatibility. Hence, G has gradually become an ideal candidate for researchers to construct novel electrochemical biosensors with high sensitivity and low detection limit. Conductive polymers are widely considered as an ideal coupling interface for constructing various types of biosensing detection systems due to their advantages of intrinsic conductivity, in situ synthesis, controllable surface properties, good biocompatibility and stability, etc. Most importantly, the composite of graphene and conductive polymer can impart G’s optical, electrical and mechanical properties to conductive polymers to improve their macroscopic performance, together with effectively increasing active sites of G to improve its electrocatalytic activity and make up for its defects in selectivity. Numerous studies have shown that PEDOT has better stability, higher conductivity and is less susceptible to oxidation than other conductive polymers. Besides, sensors modified with it could selectively detect DA without the interference of AA and UA [18,19]. In addition, the incorporation of poly (styrene sulfonate) (PSS) during the polymerization of EDOT, cannot merely greatly enhance the solubility of PEDOT in aqueous solutions, but also improve its conductivity and stability to some extent [20]. Moreover, CS, self-charged natural polymer material, was also used in this work with the aim to achieve layer-by-layer (LBL) self-assembly [21,22] modifications of PEDOT:PSS and CS–G on the surface of SPEs. Compared with other methods of preparing composite film such as electrochemical and chemical oxidation polymerization deposition, LBL has advantages of simple operation, diversified assembly materials, controllable assembly process, mild preparation conditions, low requirements to equipment and low costs, etc. [23]. According to the principle of LBL, CS with positive charges and PEDOT: PSS with negative charges, deposited alternately layer by layer under electrostatic interaction on the surface of the working electrode. Additionally, the agglomeration between G sheets can be effectively reduced by evenly distributing G to the solution of CS. With the aim of maximizing the performance of electrodes, the above three materials were combined to form nanocomposites.

As the most popular mobile devices in the world, smartphone has high-speed computing capabilities and large-capacity data storage capabilities, which are comparable with those of personal computer or laptop [24]. Moreover, it has an user-friendly interface to enable easy operation for all ages [25]. More important, it has an open-source operating system, which allows for its combination with different sensing systems to reduce cost and improve portability. With the help of the smartphone, more and more researchers have combined it with electrochemistry to develop new biomedical testing equipment for detecting biochemical substances [26,27] to support health services. Mobile health (mHealth) came into existence, based on which, people could use the related mHealth applications on smartphone to self-monitor their health conditions, such as blood pressure and blood glucose concentration [28]. Traditional detection methods require large, expensive instruments and complicated and time-consuming operations, which make it impossible to achieve rapid and real-time detection. With the advantages of a smartphone and combination of different sensors, the above electrochemical methods could be used for continuous monitoring of DA and other various biochemical substances. According to changes in response signals, variation of concentrations of biochemical substances could be reflected in time. Thus, it is of great potential to develop a smartphone-based electrochemical system for real-time monitoring of biochemical substances in the point-of-care testing (POCT) [29].

In this study, a set of smartphone-based electrochemical point-of-care testing potentiostat system was structured. Through connecting different modified electrodes, this device can be used for detection and analysis of various biomarkers. In our previous work, it was successfully applied for the detection of α-saliva amylase which was regarded as stress biomarker [30]. In this work, the practicality of this device was further researched by means of employing it for monitoring DA which was considered as emotional biomarker. It was composed of a portable potentiostat, a modified screen-printed electrode [31] as electrochemical sensor and a smartphone application (app). This detection system was employed to measure changes in electrical signals corresponding to dopamine concentration changes on the sensors. Porous nanocomposites consisting of G, CS and PEDOT: PSS were used to modify SPEs and prepared for simple electrochemical biosensors, which showed high selectivity and sensitivity to DA. The hand-held hardware device was utilized to perform electrochemical detection methods such as CV, DPV, etc. and deliver the electrical signal data. As the core of the system, the smartphone with a designed app was employed to send commands, perform calculations and display real-time data. Compared with other smartphone-based electrochemical sensing POCT system [26,32,33], the sensing detection platform designed in this work have many advantages. First, in terms of the hardware design, the commercial Arduino board equipped with analog-to-digital converter (ADC) was exploited as a microcontroller, which greatly simplified the design as a result of no extra layout of A/D conversion and microcontroller module in the detection circuit. In addition, the variety of the design for electrode interfaces allowed experimental researches via either SPE or commonly used commercial electrodes. Second, for the modification of working electrode, LBL self-assembly technology was employed instead of traditional electrochemical and chemical deposition methods, which significantly reduced the difficulty in preparing sensors with excellent performances. Finally, in terms of the app design, four detection methods were integrated and developed (square wave voltammetry (SWV) and chronoamperometry(i–t) will be applied in subsequent researches), which greatly improved the diversity in types of data that characterized properties.

## 2. Materials and Methods

### 2.1. Materials and Chemicals

DA (C_8_H_11_NO_2_), AA (C_6_H_8_O_6_) and UA (C_5_H_4_N_4_O_3_) were purchased from Sigma-Aldrich Co., LLC (Saint Louis, MO, USA). Potassium ferricyanide (K_3_[Fe(CN)_6_]), potassium phosphate (K_2_HPO_4_), potassium hydrogen phosphate (KH_2_PO_4_), sodium hydroxide (NaOH, 25%), potassium chloride (KCl), hydrochloric acid (HCl), acetic acid (CH_3_COOH), anhydrous Ethanol, ammonium persulfate (APS), ferric sulfate (Fe_2_(SO_4_)_3_) and 732 sodium-type cation exchange resin were purchased from Sinopharm Chemical Reagent Co., Ltd (Shanghai, China). All of them are analytical grade. All samples required in experiments were prepared using Milli-Q deionized water (resistivity 18 MΩ·cm@25 °C). Appropriate amounts of DA, AA and UA were dissolved in water to acquire stock solutions with a concentration of 5 mM. Serial concentrations of experimental standard solutions were freshly prepared by diluting the previously prepared stock solutions with deionized water. An amount of 0.1-M phosphate buffer solutions (PBS, pH 7.4) prepared from the mixture of K_2_HPO_4_ and KH_2_PO_4_ was used to adjust the pH balance of the electrochemical reaction system and employed with supporting electrolyte KCl (0.1 M) as reaction bottom solution. In addition, for modification of the working electrode, G (sheet diameter: 10 μm, >99.9%, Suzhou East Inspection), CS (deacetylation degree: 93%, Jinan Haidebei) and EDOT (>99.8%), PSS–Na and poly (ethyleneimine) purchased from Shanghai Aladdin Biochemical Technology Co., Ltd (Shanghai, China). were used in this work.

The SPEs used in this work, including polyethylene terephthalate (PET, with three-dimensions of 5 × 15 × 0.8 mm) and an integrated three-electrode system consisted of carbon as working, counter electrode and Ag/AgCl as reference electrode, were a kind of novel electro-analytical electrode purchased from Mxense Biotechnology Co., Ltd. (Ningbo, China).

All the above operations were carried out at ambient temperature.

### 2.2. Instruments and Apparatus

In this work, Ultrasonic cleaner (KQ-300DE, Kunshan Ultrasonic Instrument Co., Ltd. (Kunshan, China)) and High-speed refrigerated centrifuge (Z206, Hermle, Germany) were assisted in configuring the modification solution. As well, deionized water used in the preparation of analytical solutions was obtained through a pure water meter (Millipore-Q, Millipore, Burlington, MA, USA). All experiments were performed at ambient temperature.

### 2.3. Design of Detection Circuit and App

**Figure 1:**
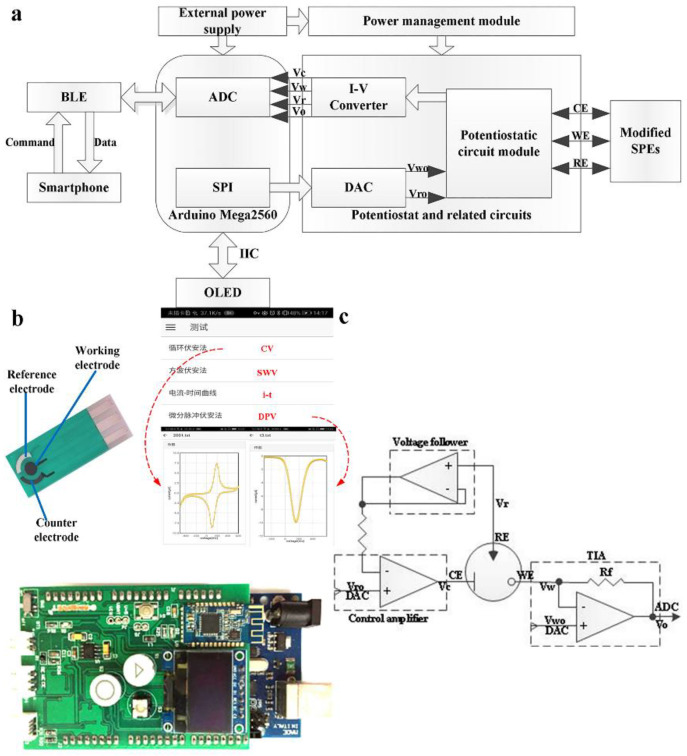
(**a**) The schematic diagram of the smartphone-based electrochemical biosensing system; (**b**) The images of components of the system; (**c**) The schematic diagram of potentiostat and I–V converter.

The whole detection circuit is mainly composed of a microcontroller module, a D/A module, a potentiostat circuit module, a Bluetooth module, an OLED and a power management module. As shown in Figure 1a, the microcontroller module (Arduino Mega 2560, JKSS Co., Ltd. Rochester, Kent, United Kingdom) controls an external digital-to-analog converter (DAC, LTC1655I, Linear) by using a serial peripheral interface (SPI) communication to apply a specific analog potential signal to external electronic analysis electrodes (SPEs). A potentiostat circuit module composed of three amplifiers (MCP6072-E/SN, Microchip) is utilized to maintain a constant and stable output between the working electrode and the reference electrode, and automatically adjusts the potential shift occurring during the electrochemical reaction. The loop current generated by the reaction between the working electrode and the counter electrode is converted into an analog potential signal through a microcurrent detecting circuit including a current–voltage converting circuit and an amplifying circuit. The digital signal that it can acquire and store is then obtained via an analog-to-digital converter (ADC) on the microcontroller. The electrical signals collected by the microcontroller are sent to the upper smartphone app for reception through the Bluetooth Low Energy Module (BLE, Shenzhen Keyian Microelectronics Co., Ltd. Shenzhen, China). Smartphone app uses the Bluetooth communication method, on one hand, to send an instruction to the detection circuit to select a detection method and set initial parameters; on the other hand, to receive and process digital signals from the detection circuit. Furthermore, the OLED together with buttons installed on the detection circuit can also set the detection method and parameters through the pre-compiled code. The entire detection circuit is externally connected to a USB power supply or a dry battery pack and uses a power management module to achieve continuous and effective power supply.

As shown in Figure 1b, the whole self-designed detection system contains three main parts: a modified SPE as electrochemical sensor, a hand-held detector and a smartphone with a designed application. The detector circuit was used to generate excitation voltage, measure the consequent current on the sensors and deliver electrical signal data via the BLE module. The designed application can perform four detection modes: cyclic voltammetry (CV), square wave voltammetry (SWV), chronoamperometry and differential pulse voltammetry (DPV). It was employed to send commands with initial parameters: voltage range, scan rate, pulse width, pulse period, pulse amplitude, calculate electrical signal based on the data and plot signal curve for real-time display on the smartphone.

As shown in Figure 1c, the potentiostat together with the I–V converter is composed of three operational amplifiers. Among them, one serves as a control amplifier to continuously compare the input potential with that between the working and reference electrodes and maintain its value as a programmed value. Moreover, it can also supply unlimited output current to activate and maintain the electrochemical reaction. The other one acts as a potential follower to reduce the potential change resulted from the current flowing through the reference electrode. The rest one forms a resistive feedback transimpedance amplifier (TIA) with a variable feedback resistor, in which the potential drop on the feedback resistor was measured to indirectly obtain the reaction current of different orders of magnitude in the electrochemical system through the following formula:

(1) $$I=\frac{V_{o}-V_{w}}{R_{f}},$$

### 2.4. Modification of SPEs

The solution for modification included G and CS and PEDOT incorporated with PSS (PEDOT: PSS). The solution was prepared as the following steps. (A) Preparation of G solution: 1 g G was accurately measured and dissolved in deionized water, followed by centrifugation. Then, the supernatant of it was removed and ultrasonically dissolved for 30 min to obtain a well-distributed graphene suspension. (B) Preparation of CS solution: 1 g CS was accurately measured and dissolved in an appropriate amount of CH_3_COOH (2%) with continuous stirring, and then diluted with deionized water to obtain 100 mL CS solution (1%). (C) Preparation of PEDOT:PSS solution: First, EDOT and PSS–Na were accurately measured at a ratio of 1:3 [34]. Second, PSS–Na was dissolved in deionized water with continuous stirring until a yellow transparent solution was formed. Third, EDOT was gradually added with continuous stirring. HCl was used to maintain the pH of the solution in the range of 2–3. Then, a mixture of APS as oxidant and Fe_2_(SO_4_)_3_ as catalyst was added to the above solution and waited until it turned to dark blue. Finally, 732 sodium-type cation exchange resins were used to purify PEDOT:PSS–Na and ultrasonically processed for 30 min to obtain a well-distributed PEDOT:PSS suspension [35]. All the above solutions were stored without exposure to light at 4 °C.

**Figure 2:**
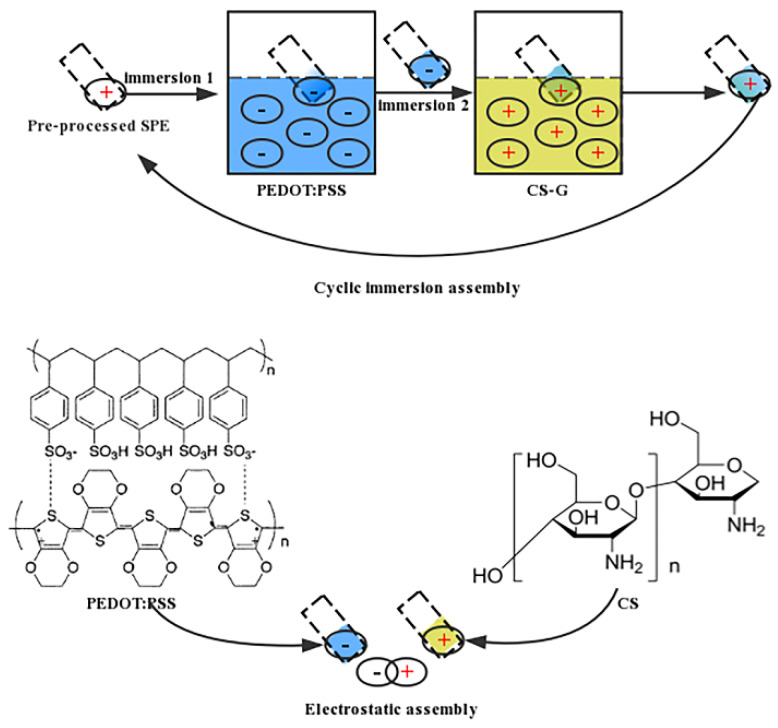
The process of layer-by-layer electrostatic self-assembly.

Prior to the modification, the SPEs were washed with a mixture of deionized water and alcohol and sonicated for 15 min. In addition, to make the above modification materials a better attach to the surface of working electrode, the SPE whose counter and reference electrode were temporarily covered with hydrophobic double-sided tape (or waxing), was immersed in poly (ethyleneimine) (30 mg/mL) as cationic surface finishing agent with heating at 75 °C in the water bath for 1 h. After that, the electrode was positively charged. Finally, as shown in Figure 2, the pre-charged SPE was cyclically immersed with four and a half times in PEDOT: PSS suspension and sonicated graphene/chitosan (CS–G) to deposit them alternately layer by layer on the surface of the working electrode. According to some related research reports [36], the conductivity of the electrode will be gradually improved through increasing the amount of self-assembled layers within a certain range, as a consequence of which, the current responses will be maximized as much as possible. It will hinder electronic transfer once the self-assembled film is too thick. In the end, a simple electrochemical sensor modified with four layers of CS–G and five layers of PEDOT: PSS for dopamine detection was successfully prepared. As well, the above operations were repeated to prepare a plurality of modified SPEs in advance and stored without exposure to light at 4 °C for subsequent experiments.

After the modification, CV and DPV were performed to investigate the changes in the electrochemical behavior of the SPE duo to different modification materials in the presence of K_3_ [Fe(CN)_6_] or a mixture of DA, AA, UA in 0.1-M PBS (pH: 7.4, 0.1-M KCl). CV was carried at the rate of 50 mV/s with a potential range varying from −500 to + 600 mV, while DPV was performed from −100 to +560 mV (50 mV pulse amplitude, 2 mV step potential, 0.05 s pulse width).

### 2.5. Electrochemical Characterization of Modified SPEs

The electrochemical properties of the single/composite modified SPEs were further investigated through CV at a range of rates (10 mV/s–100 mV/s) by dropping onto the SPEs 50 μL of 10 μM DA or a mixture of 10 μM DA, 10 μM AA, 10 μM UA in presence of 0.1-M PBS (pH: 7.4, 0.1-M KCl), with a potential range scanned from −500 to +600 mV. The current peak value was taken as the electrochemical signal and plotted against the related deformation parameters of the scan rate. The obtained curve was fitted by using Origin9 software.

### 2.6. Dopamine Detection

The calibration curve of dopamine was acquired by dropping various DA solutions at different concentrations ranging from 0.05 to 70 μM in the presence of 30 μM AA and 30 μM UA in 0.1-M PBS (pH: 7.4, 0.1-M KCl) onto the single/composite modified SPE. DA, AA and UA, as electroactive compounds, were oxidized and detected using DPV with a potential range varying from −100 to +560 mV (50 mV pulse amplitude, 2 mV step potential, 0.05 s pulse width). The current peak value was taken as the electrochemical signal and plotted against DA concentration. The obtained curve was fitted by using Origin 9 software.

## 3. Results and Discussion

### 3.1. Electrochemical Characterization of SPEs Modified with Different Materials

**Figure 3:**
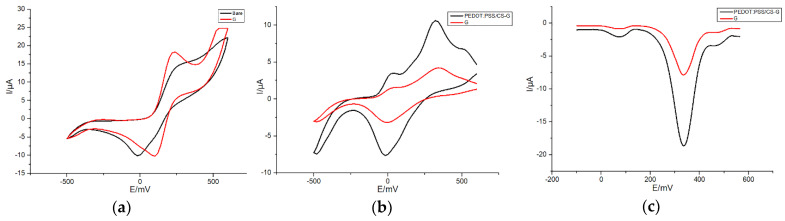
(**a**) Cyclic voltammetry (CV) of 50 μM K_3_ [Fe(CN)_6_] in 0.1-M PBS (pH: 7.4, 0.1-M KCl) at bare and modified screen-printed electrode (SPE) (G/SPE); (**b**) CVs of 20 μM dopamine (DA), 20 μM ascorbic acid (AA) and 20 μM uric acid (UA) in 0.1-M phosphate buffer solutions (PBS) (PH: 7.4, 0.1-M KCl) at poly(3,4-ethylenedioxythiophene) (PEDOT):PSS/CS–G/SPE and G/SPE; (**c**) differential pulse voltammetry (DPV) responses of 30 μM DA, 30 μM AA and 30 μM UA in 0.1-M PBS (pH: 7.4, 0.1-M KCl) at PEDOT:PSS/CS–G/SPE and G/SPE. Detection parameters: CV—potential range from −500 to +600 mV, 50 mV/s scan rate; DPV—potential range from −100 to +560 mV, 50-mV pulse amplitude, 2-mV step potential, 0.05-s pulse width.

First, changes of the SPE’s electrochemical behavior resulted from G were investigated by means of CV at the rate of 50 mV/s with a potential range varying from −500 to +600 mV in presence of a redox probe [Fe(CN)_6_] ^3−/4−^ in 0.1-M PBS (pH: 7.4, 0.1-M KCl). As shown in Figure 3a, the current response measured on the SPE was significantly enhanced after that graphene was modified onto the working electrode. According to the Randles–Sevcik equation, under the same reaction conditions, the redox peak current of CV depends on the electrochemically active area of the modified electrode. It could be observed in Figure 3a that the electroactive area of bare SPE increased to some extent after G was modified onto it. Furthermore, after PEDOT: PSS/CS was modified onto the G/SPE (Figure 3b), the electroactive area of the electrode enlarged significantly. This phenomenon is probably associated with the large specific surface area of graphene and conductive polymer. Meanwhile, it also demonstrates that the conductive polymer effectively improved the surface catalytic activity of graphene, which greatly promoted the electrocatalytic reaction of DA onto the working electrode.

Then, electrochemical sensing properties of G/SPE and PEDOT: PSS/CS–G/SPE were investigated through CV and DPV, respectively to detect DA in the presence of AA and UA in 0.1-M PBS (pH: 7.4, 0.1-M KCl). As shown in Figure 3b, three CV anodization peaks of DA, AA and UA were clearly distinguished on PEDOT: PSS/CS–G/SPE. The oxidation peaks appeared at about 40 mV (AA), 320 mV (DA) and 500 mV (UA). The peak separations of PEDOT: PSS/CS–G/SPE were 280 mV (AA and DA), 180 mV (DA and UA) and 460 mV (AA and UA). The ΔEp (AA and DA) value was significantly larger than that in the case of Pt/GSPE in an earlier report [37]. Correspondingly, compared with G/SPE, the PEDOT: PSS/CS–G/SPE offers a higher anodization peak current and improved voltammetric peak separation for the measurement of the three analytes. Hence, the PEDOT: PSS/CS–G/SPE supplies superior performance in terms of responsiveness and selectivity. Furthermore, only a significant reduction peak appeared on G/SPE and PEDOT: PSS/CS–G/SPE, respectively, which proved that only the electrochemical catalysis reaction of DA was reversible. As shown in Figure 3c, three different and determined anodization peaks were clearly distinguished on PEDOT: PSS/CS–G/SPE at about 72 mV (AA), 336 mV (DA) and 460 mV (UA). Peak separations were 264 mV (AA and DA), 124 mV (DA and UA) and 388 mV (AA and UA). Similarly, the oxidation peak current at PEDOT: PSS/CS–G/SPE was obviously higher than that obtained at G/SPE, which demonstrated higher electrocatalytic activity than G/SPE towards the electrochemical reaction of DA. Furthermore, it is important to notice that the ΔEp (AA and DA) of PEDOT: PSS/CS–G/SPE is wider than that of G/SPE. The PEDOT: PSS/CS–G/SPE thus illustrated better selectivity to DA.

### 3.2. Scan Rate Dependence of Peak Current on Modified SPEs

**Figure 4:**
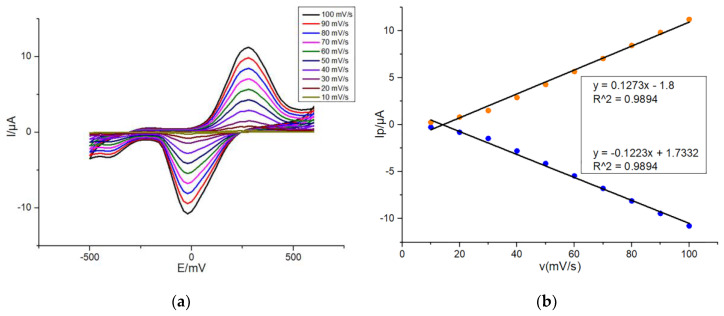
(**a**) CVs of 10 μM DA in 0.1-M PBS (pH: 7.4, 0.1-M KCl) at G/SPE with a scan rate from 10 to 100 mV/s in 10 mV/s steps; (**b**) Plots of peak currents of DA oxidation and reduction versus scan rates. Detection parameters: potential range from −500 to +600 mV.

In order to further characterize the electrochemical redox of DA on G/SPE and PEDOT: PSS/CS–G/SPE, firstly, CV was performed by dropping onto G/SPE 50 μL of 10 μM dopamine in presence of 0.1-M PBS (pH: 7.4, 0.1-M KCl) with a scan rate varying from 10 to 100 mV/s in 10 mV/s steps. As shown in Figure 4a, redox peak currents measured on G/SPE were positively correlated with scan rates. Moreover, with increase in the scan rate, the anodic oxidation peak potential and the cathodic reduction peak potential of DA slightly shifted positively and negatively, respectively. It fully proved that the electrochemical reaction of DA on the modified SPE was reversible. As shown in Figure 4b, oxidation and reduction peak currents of DA were almost linearly related to scan rates, respectively during increasing the scan rate from 10 to 100 mV/s, which indicated that the reaction of DA on G/SPE was an adsorption-controlled process. The possible reason was that the DA could not completely diffuse in the modification film because of the fast scan rate [38]. Hence, the whole electrochemical reaction process of DA on G/SPE was influenced by the adsorption effect.

**Figure 5:**
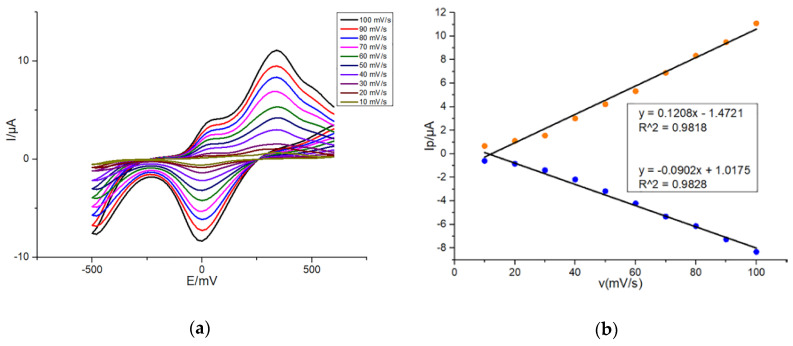

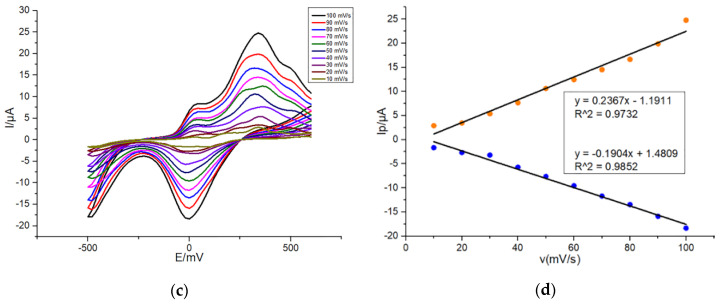
CVs of 10 μM DA, 10 μM AA and 10 μM UA in 0.1-M PBS (pH: 7.4, 0.1-M KCl) at G/SPE (**a**) and PEDOT:PSS/CS–G/SPE (**c**) with a scan rate from 10 to 100 mV/s in 10 mV/s steps; (**b**,**d**) plots of peak currents of DA oxidation and reduction versus scan rates. Detection parameters: potential range from −500 to +600 mV.

Then, CV was performed onto G/SPE and PEDOT: PSS/CS–G/SPE, respectively to detect dopamine in the presence of AA and UA in 0.1-M PBS (pH: 7.4, 0.1-M KCl) with a scan rate varying from 10 to 100 mV/s in 10 mV/s steps. Comparing Figure 5a,c, redox peak currents at PEDOT:PSS/CS–G/SPE were significantly higher than those obtained at G/SPE in the case of same scan rates. Selective and simultaneous determination of AA, DA and UA is demonstrated by the distinct difference between the anodic peaks of them at PEDOT: PSS/CS–G/SPE (Figure 5c), which were clearer than the oxidation peaks of them at G/SPE (Figure 5a). Moreover, the separation between the redox peaks of ΔE for DA-AA, UA-DA and UA-AA is improved compared with those reported for graphene-based electrodes [39]. Accordingly, it was obvious that not only PEDOT:PSS/CS–G/SPE demonstrated higher electrocatalytic activity to the electrochemical reaction of DA and outstanding reversibility, but also it illustrated better selectivity to determination of DA. Specifically, the superior performance of PEDOT:PSS/CS–G/SPE for detecting DA, on one hand, depends on the good conductivity of graphene and conductive polymers; on the other hand, it can be attributed to the selectivity of negatively charged PEDOT:PSS for cationic neurotransmitter–DA without interference from anionic AA/UA [40]. From Figure 5b,d, the conclusion similar to Figure 4b could be obtained that in the case of coexistence of DA, AA and UA, the whole electrochemical redox reaction process of DA on SPEs modified with different materials was also influenced by the adsorption effect.

On behalf of reducing the background signal response and improve the signal-to-noise ratio (S/N), 50 mV/s was used as the optimal scan rate in subsequent experiments.

### 3.3. Dopamine Calibration Curve

**Figure 6:**
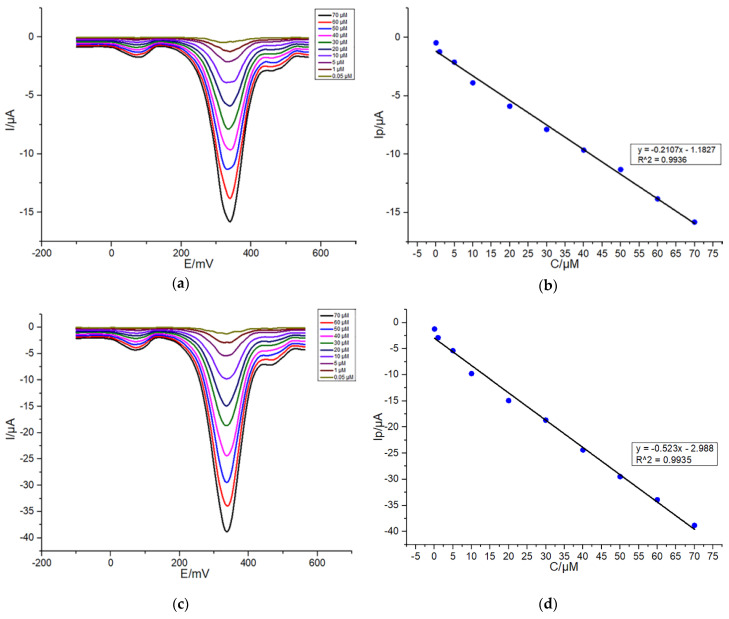
DPV responses of varying concentrations of DA from 0.05 to 70 μM in the presence of a mixture of 30-μM AA and 30-μM UA in 0.1-M PBS (pH: 7.4, 0.1-M KCl) at G/SPE (**a**) and PEDOT:PSS/CS–G/SPE (**c**); (**b**,**d**) plots of peak currents of DA oxidation versus DA concentrations. Detection parameters: potential range from −100 to +560 mV, 50-mV pulse amplitude, 2-mV step potential, 0.05-s pulse width.

To evaluate the performance of the self-designed electrochemical sensing system for selective detection of dopamine, DPV was performed to record increases in DA oxidation peak currents due to the increase of dopamine concentration ranging from 0.05 to 70 μM and fit two calibration curves, by dropping onto G/SPE and PEDOT: PSS/CS–G/SPE 50 μL of different concentrations of DA in presence of a mixture of 30 μM AA and 30 μM UA in 0.1-M PBS (pH: 7.4, 0.1-M KCl). As shown in Figure 6a,c, as DA concentration increasing, oxidation peak currents corresponding to constant concentrations of AA and UA only slightly increased irregularly, suggesting that the addition of AA and UA does not interfere with the detection of DA. The concentration range selected in this work fits well with the normal blood concentration range for DA [41]. As a consequence, this self-designed electrochemical potentiostat can be applied for DA detection in actual samples. As shown in Figure 6b,d, DA oxidation peak currents measured on G/SPE and PEDOT:PSS/CS–G/SPE were linearly related to DA concentration ranging from 0.05 to 70 μM.

From Figure 6d, a linear relationship was obtained (y = −0.523x − 2.988) with a regression value of 0.9942. The detection sensitivity (M) of PEDOT: PSS/CS–G/SPE was about 0.52 ± 0.01 μA/μM, which was higher than that of G/SPE (0.21 ± 0.01 μA/μM, Figure 6b). Moreover, the PEDOT: PSS/CS–G/SPE displayed higher sensitivity towards DA compared with AA and UA of the same concentrations as the DA did. This can be attributed to that PEDOT: PSS with negative charge attracts the positively charged DA and contributes to the reaction of gain and loss of electrons for it on the surface of the electrode, while repelling AA and UA with negative charge [42]. The limit of detection (LOD), calculated according to the formula 3 Sb/M (S/N = 3), where M is the slope of the calibration curve and Sb is the standard deviation of the blank signal, was about 0.29 μM. The wider linear range and lower detection limit using our PEDOT: PSS/CS–G/SPE based on self-designed detection system are acquired as compared with some literature reports [15,17,43,44,45,46] summarized in Table 1.

## 4. Conclusions

In this work, we designed and developed an electrochemical sensing system for fast and real-time detection of DA. The LBL was applied for modification of SPE used as a sensor by alternately depositing PEDOT, CS and G onto the working electrode. By combining the prepared modified sensor with the self-designed electrochemical potentiostat, highly selective detection of DA in the presence of interfering substances such as AA and UA was achieved. In addition, a good linear relationship between the DA oxidation peak current values and varying DA concentrations ranging from 0.05 to 70 μM, with a detection sensitivity of 0.52 ± 0.01 μA/μM and a limit of detection of 0.29 μM, was obtained. Due to limited time, tests and analysis with real blood or urine samples were not employed in this work. Hence, the practical application worth of this electrochemical sensing system will be evaluated through following further investigations.

Considering the good selectivity, sensitivity of the modified sensor for DA, and the simplicity of its preparation process, as well as the portability and low cost of the detection device, it is promised that the self-designed electrochemical sensing system will be widely used for DA detection in POCT analysis after more experimental explorations together with adjustment and optimization for architectures of software and hardware in this system.

## Figures and Tables

**Table 1:** Comparison of our PEDOT:PSS/CS–G/SPE using a self-designed detection system with other modified electrodes using various methods reported in the literature for the determination of DA.

Modified Electrodes	Linear Range (μM)	Detection Limit (μM)
PEDOT:PSS/CS–G/SPE (Our work)	0.05–70	0.29
SDS/SPCE [43]	1–100	0.37
Fe_3_O_4_@Au-Gr/GCE [44]	0.05–50	0.65
Au/RGO/GCE [45]	6.8–41	1.4
RuO_2_/RGO/GCE [46]	3–200	875
AuNPs@PANI/GSPE [17]	1–100	0.86
PANI-rGO-NF [15]	0.05–180	0.024
